# Ångstrom-scale gold particles loaded with alendronate via alpha-lipoic acid alleviate bone loss in osteoporotic mice

**DOI:** 10.1186/s12951-024-02466-9

**Published:** 2024-04-30

**Authors:** Weihang Gao, Jiao Jiao Li, Jingyu Shi, Hongbing Lan, Yuanyuan Guo, Dehao Fu

**Affiliations:** 1 Department of Orthopaedics, Shanghai Sixth People’s Hospital, Shanghai Jiao Tong University School of Medicine, Shanghai, 200233 P. R. China; 2 Liyuan Hospital, Tongji Medical College, Huazhong University of Science and Technology, Wuhan, 430077 China; 3 School of Biomedical Engineering, Faculty of Engineering and IT, University of Technology Sydney, Sydney, NSW 2007 Australia; 4 Tongji School of Pharmacy, Huazhong University of Science and Technology, Wuhan, 430030 China; 5 Hubei Key Laboratory of Metabolic Abnormalities and Vascular Aging, Huazhong University of Science and Technology, Wuhan, 430077 China

**Keywords:** Osteoporosis, Gold particles, Ångstrom, Alendronate, Bone targeting, Alpha-lipoic acid

## Abstract

Osteoporosis is a highly prevalent metabolic disease characterized by low systemic bone mass and deterioration of bone microarchitecture, resulting in reduced bone strength and increased fracture risk. Current treatment options for osteoporosis are limited by factors such as efficacy, cost, availability, side effects, and acceptability to patients. Gold nanoparticles show promise as an emerging osteoporosis therapy due to their osteogenic effects and ability to allow therapeutic delivery but have inherent constraints, such as low specificity and the potential for heavy metal accumulation in the body. This study reports the synthesis of ultrasmall gold particles almost reaching the Ångstrom (Ång) dimension. The antioxidant alpha-lipoic acid (LA) is used as a dispersant and stabilizer to coat Ångstrom-scale gold particles (AuÅPs). Alendronate (AL), an amino-bisphosphonate commonly used in drug therapy for osteoporosis, is conjugated through LA to the surface of AuÅPs, allowing targeted delivery to bone and enhancing antiresorptive therapeutic effects. In this study, alendronate-loaded Ångstrom-scale gold particles (AuÅPs-AL) were used for the first time to promote osteogenesis and alleviate bone loss through regulation of the WNT signaling pathway, as shown through in vitro tests. The in vivo therapeutic effects of AuÅPs-AL were demonstrated in an established osteoporosis mouse model. The results of Micro-computed Tomography, histology, and tartrate-resistant acid phosphatase staining indicated that AuÅPs-AL significantly improved bone density and prevented bone loss, with no evidence of nanoparticle-associated toxicity. These findings suggest the possible future application of AuÅPs-AL in osteoporosis therapy and point to the potential of developing new approaches for treating metabolic bone diseases using Ångstrom-scale gold particles.

**Supplementary Information:**

The online version contains supplementary material available at 10.1186/s12951-024-02466-9.

## Background

Osteoporosis is a debilitating metabolic bone disease characterized by systemic loss of bone mass and strength, deterioration of bone microarchitecture, and significantly increased fracture risk. It is the most prevalent bone-related disease worldwide, affecting over 200 million individuals and most frequently occurring in postmenopausal women [1]. At present, clinical drug therapies of osteoporosis mainly include bisphosphonates, calcitonin, estrogen, raloxifene, and RANK ligand inhibitors, as well as a combination of vitamin D and calcium supplements. However, most of these drugs do not offer a long-term solution due to their limitations and side effects [2, 3]. For instance, their systemic administration necessitates high doses and frequent use to achieve moderate effects on alleviating bone loss, which can result in serious adverse effects [4]. Meanwhile, certain drugs such as denosumab, raloxifene and RANK ligand inhibitors may have higher treatment costs that need to be considered. Thus, developing new types of targeted drug therapies with high efficiency and low toxicity remains a top priority for the treatment of osteoporosis.

Nanomaterials have gained significant attention in recent years as therapeutic delivery vehicles due to their unique physicochemical properties, such as ultrasmall size, large ratio of surface area to mass, and facile surface modification [5]. Among various metal nanoparticles, gold nanoparticles (AuNPs) stand out for biomedical applications due to their exceptional stability, high in vivo safety and efficiency, and minimal release of metal ions [6]. Recent evidence suggests that AuNPs can not only function as nanocarriers for transporting therapeutic drugs but also act as osteogenic agents, as they possess the ability to promote osteogenic differentiation and mineralization [7–9]. Despite their unique advantages, current types of AuNPs experience some major limitations as nanocarriers for treating bone diseases. Most AuNPs range in size from 5 to 100 nm and are challenging to eliminate from the body due to their comparatively large size on the nanoscale. These larger AuNPs are prone to being trapped by the reticuloendothelial system (RES), leading to low uptake in bone tissue upon systemic administration, followed by their inevitable accumulation in major organs, such as the liver and spleen. Reducing the particle size may help to avoid RES absorption [10]. Recent studies have reported that AuNPs with sizes below 20 nm can effectively escape RES absorption, enabling good cellular uptake [11]. However, these particle sizes still exceed the renal clearance barrier of 5.5 nm, raising concerns about long-term accumulation and toxicity to the kidneys or other parts of the renal system [12, 13]. A potential solution to this problem is to optimize the morphology and dimensions of nanomaterials, such as by synthesizing ultrasmall-sized gold particles. Recently, metal nanoparticles smaller than 3 nm have been increasingly studied in various biomedical applications, including bioimaging, drug delivery, and photothermal treatment [14–16]. These ultrasmall nanoparticles can be efficiently eliminated from the body due to their minute size [17]. Ultrasmall gold particles have been used for a variety of applications, but their capabilities as a drug delivery vehicle are just beginning to be explored [18, 19], while studies involving bone tissue remain limited. Ultrasmall gold particles may address the shortcomings of traditional AuNPs for drug delivery to bone tissue, where an excessively high concentration of gold particles would lead to cell death, but low concentrations might be insufficient to achieve a significant therapeutic effect. To reduce the particle dosage needed for systemic administration while still preserving drug delivery efficiency, one idea is to enhance gold particle absorption and distribution in bone tissue by improving their targeting ability. This may be achieved by considering the surface chemical modification of ultrasmall gold particles.

Alpha-lipoic acid (LA) is a robust antioxidant and serves as a substrate for the pyruvate dehydrogenase complex within the mitochondria of renal, hepatic, and cardiac tissues. Notably, LA plays a pivotal role in mitigating the detrimental effects of oxidative stress and apoptosis by scavenging reactive oxygen species (ROS) and protecting various cell types [20]. There is some evidence to suggest that LA has potential therapeutic benefits for the treatment of osteoporosis [21, 22]. Recent studies also indicate that the combination of natural antioxidants such as LA with gold nanomaterials may mitigate potential nanomaterial-associated toxicity [23]. These findings give rise to the idea of using LA to functionalize the surface of ultrasmall gold particles, as ligands with at least two binding sites, such as LA and its derivatives, have been shown to significantly enhance the stability of metal and semiconductor nanocrystals [24]. Moreover, the hydrophilic carboxyl group in LA can serve as an anchor for immobilizing biological molecules through either electrostatic interactions or covalent bonding [25].

Using LA as a bridging molecule opens up the exciting potential to immobilize osteoporosis drugs to the surface of ultrasmall gold particles. Among the drug candidates, bisphosphonates such as alendronate (AL) belong to a class of synthetic compounds structurally related to pyrophosphate. Their strong affinity for hydroxyapatite (HA) crystals results in their rapid localization on bone surfaces after peroral or intravenous administration [26]. Bisphosphonates have shown potent bone-preserving effects in other types of bone diseases, such as osteogenesis imperfecta, where their use has been reported to enhance mobility, reduce fracture occurrence, and alleviate bone pain [27]. Their mechanisms of action include regulating the proliferation and differentiation of osteoblasts, initiating survival signaling pathways that contribute to bone homeostasis and exerting antiresorptive effects [28]. Considering the abovementioned complementary advantages of individual design components, we propose that the incorporation of AL through LA into ultrasmall gold particles may present an innovative strategy for the treatment of osteoporosis.

**Fig. 1:**
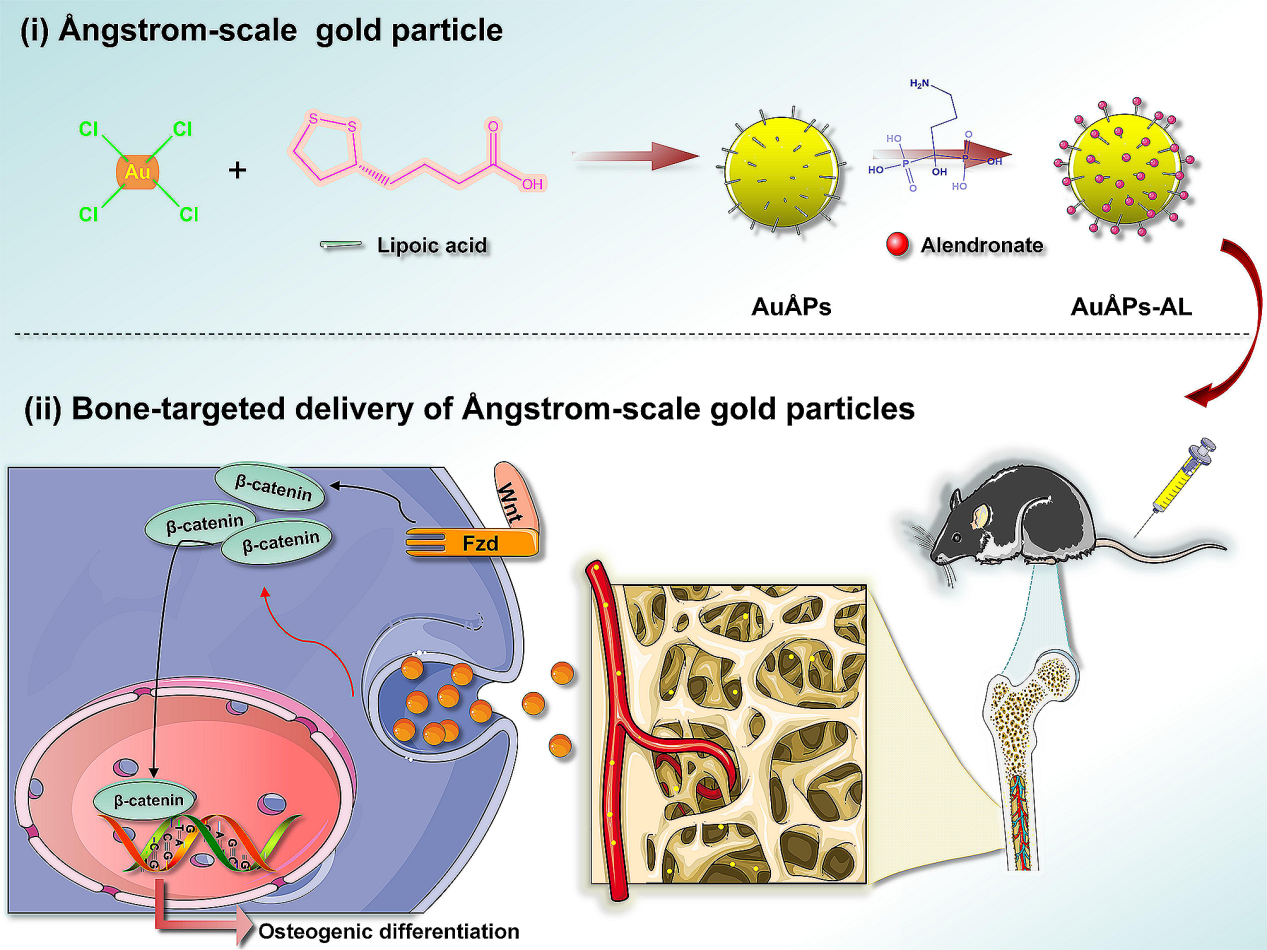
Preparation of AuÅPs-AL and their therapeutic mechanism of action

This study reports for the first time the successful synthesis of ultrasmall gold particles reaching the Ångstrom (Ång; one-tenth of a nanometer) dimension, with bone-targeting capability conveyed through AL loaded onto the particle surface through LA. Ångstrom-scale gold particles (AuÅPs) were first synthesized by utilizing LA as a dispersant and stabilizer, after which the LA functioned as a linker to conjugate AL molecules onto the surface of AuÅPs to provide bone-targeting effects. The preparation process and related mechanisms are illustrated in Fig. 1. These Ångstrom-scale gold particles (AuÅPs-AL) exhibited excellent stability and biocompatibility, minimal toxicity and great ability for in vivo bone targeting. The molecular mechanisms by which AuÅPs-AL mediated osteogenesis included regulating osteoblast differentiation and inhibiting osteoclast formation, partly through the WNT signaling pathway. Intravenously administered AuÅPs-AL in an ovariectomy (OVX)-induced osteoporosis mouse model demonstrated significantly improved bone density and mitigation of bone loss, suggesting a protective effect in osteoporosis. These novel AuÅPs-AL represent a promising therapeutic candidate for osteoporosis and point to the potential of exploring the surface modification of AuÅPs to open up new applications in tissue engineering for bones and other organs.

## Results and discussion

### Synthesis and characterization of AuÅPs-AL

**Fig. 2:**
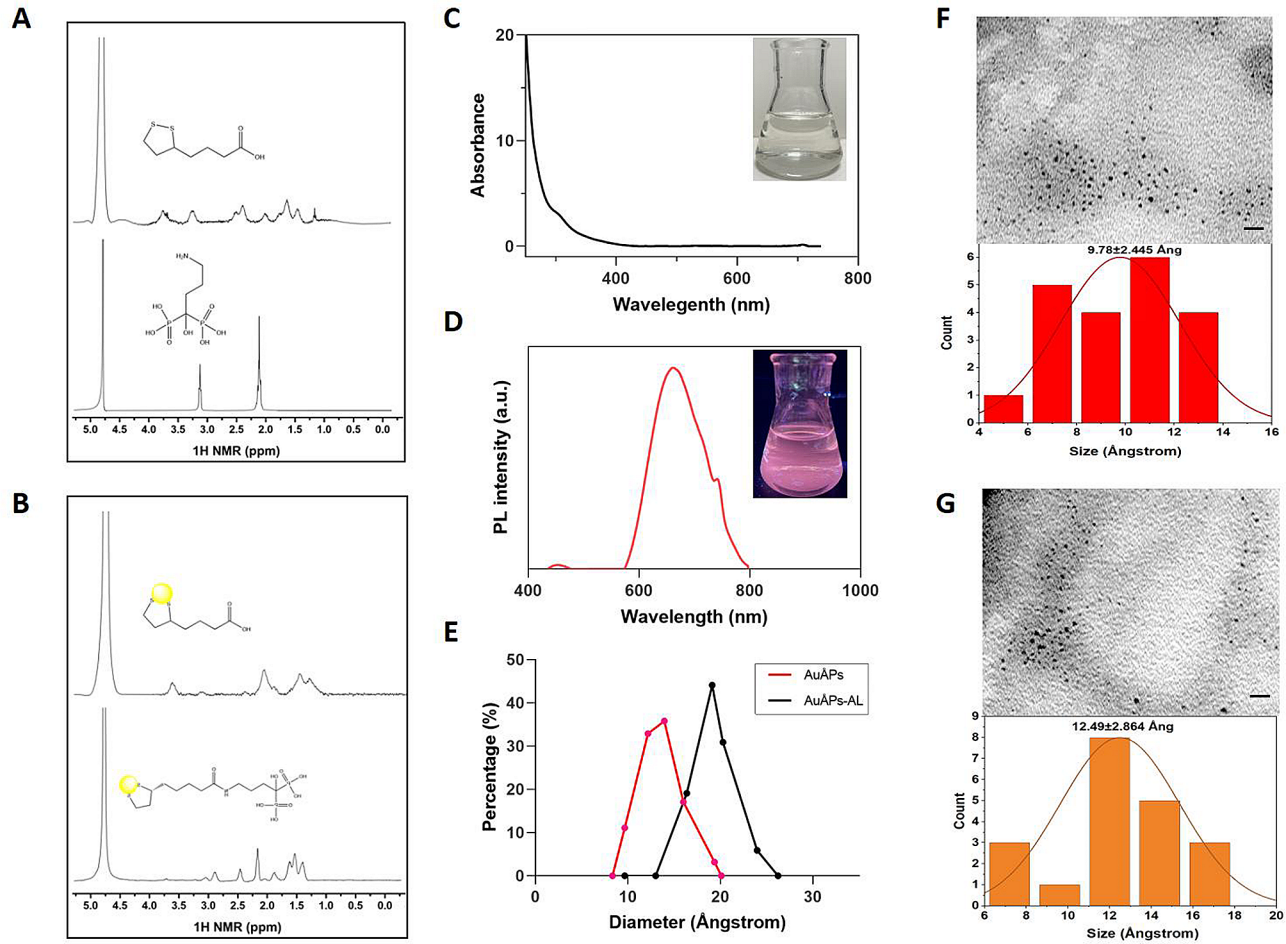
Characterization of the synthesized Ångstrom-scale gold particles with (AuÅPs-AL) or without (AuÅPs) alendronate loading. **A **^1^H-NMR spectra of free lipoic acid (top) and AL (bottom). **B **^1^H-NMR spectra of dialyzed AuÅPs (top) and AuÅPs-AL (bottom) in D_2_O. **C** Photographs of the AuÅPs-AL solution under visible light and UV light. **D** Fluorescence excitation and emission spectra of AuÅPs-AL (680 nm). **E** DLS spectra of the as-prepared AuÅPs-AL (black line) and AuÅPs (red line). **F** and **G** TEM images and core size distribution of the synthesized AuÅPs and AuÅPs-AL, respectively

AuÅPs-AL were synthesized through a multistep synthesis process, as illustrated in Fig. 1. AuÅPs were first formed by reacting an aqueous solution of chloroauric acid with LA under alkaline conditions with continuous heating. During synthesis, the disulfide groups of LA attach to the gold surface, providing stabilization to the gold particles, while the free carboxylic acid groups allow for particle dispersion in water. The chemical structure of AuÅPs was confirmed through proton nuclear magnetic resonance (NMR) analysis. For reference, the one-dimensional ^1^H-NMR spectrum and molecular structure of free lipoic acid were included (Fig. 2A), which were the same as in other reports [29]. Acid-terminated AuÅPs were subsequently activated using N-(3-dimethylaminopropyl)-N’-ethylcarbodiimide/N-hydroxysuccinimide (EDC/NHS) and bound to AL, which possesses an amino group. The AL yielded a spectrum characterized mainly by two broad singlets centered at 2.06 and 3.09 ppm (Fig. 2A). The ^1^H-NMR spectrum of the AuÅPs-AL conjugate contained a prevalence of AuÅPs signals (Fig. 2B), with the presence of AL indicated by a broad peak centered at 1.62 ppm [30, 31]. The ^1^H-NMR spectra provided evidence for the successful synthesis of AuÅPs-AL.

The purified AuÅPs-AL solution was a stable clear liquid at room temperature, which showed a light yellow color under daylight (Fig. 2C) and emitted intense red fluorescence under 365 nm ultraviolet (UV) light. The AuÅPs-AL showed a fluorescence emission peak at 670 nm (Fig. 2D). Consistent with previous findings, gold nanoparticles smaller than 3 nm belong to the “Ångstrom dimension”, exhibiting a very light color in solution and displaying characteristic fluorescence wavelengths under UV light irradiation [32]. Ultrasmall AuNPs with discrete sizes do not exhibit the characteristic strong surface plasmon resonance (SPR) absorption peak typically observed at approximately 500–550 nm [33]. Most ultrasmall AuNPs are composed of several to hundreds of gold atoms and exhibit significant fluorescence emission in the near-infrared to visible light range [34, 35], which is comparable to the Fermi wavelength of electrons [36].

The functionalized AuÅPs-AL were characterized using dynamic light scattering (DLS) through a particle size analyzer that assessed the size distribution of gold particles in solution. The uniformity and stability of the synthesized gold particles are critical for their potential biomedical applications. The hydrodynamic size of AuÅPs-AL was approximately 20 Ång (Fig. 2E, black line), slightly larger than the size of AuÅPs. The size determined by DLS is typically larger than that determined by transmission electron microscopy (TEM) because DLS measures the hydrodynamic diameter, including the particles themselves and an electric dipole layer that adheres to the particle surface in solution. In contrast, TEM images directly show the particle diameter, providing more precise measurements. As revealed by TEM, the AuÅPs displayed a spherical or ellipsoidal morphology and a relatively uniform particle size distribution over a narrow range, with an average particle size of 9.8 ± 2.4 Ång (Fig. 2F), consistent with previous reports [24]. AuÅPs-AL displayed a similar shape to AuÅPs, with a larger average diameter of 12.9 ± 2.9 Ång (Fig. 2G), and were uniformly dispersed with negligible aggregation. Following immersion for over 48 h in various solutions, including ultrapure water, saline buffer, Dulbecco’s modified Eagle medium (DMEM), and α-modified Eagle medium (α-MEM), AuÅPs-AL showed no obvious change in fluorescence properties. These results suggest the excellent stability of AuÅPs-AL under physiological conditions.

Functionalized nanoscale gold particles (AuNPs-AL) were also synthesized for comparison purposes. The citrate-stabilized AuNPs were synthesized using a modified Frens method [37]. Subsequently, a ligand exchange reaction was conducted to replace the citrate molecules with lipoic acid. The synthesized AuNPs, before and after the ligand-exchange reaction, were characterized using UV-visible spectroscopy to evaluate their functionalization with LA. AuNPs exhibit SPR, the collective oscillation of electrons in the conduction band with incident light of a specific wavelength. This behavior causes AuNPs to absorb light within the wavelength range of 500 to 600 nm, depending on their size and shape. Consequently, any modification to the surface of AuNPs leads to a shift in the plasmon band, which is detectable through UV-Vis spectroscopy. An increase in AuNP size, whether through binding with ligands or aggregation, leads to a redshift of the plasmon band [38]. Fig. S1 displays the optical absorption spectra of the gold nanoparticles before and after LA modification, where the surface plasmon band showed a slight redshift attributed to the binding of LA. This observation indicated the replacement of citrate molecules with LA through the ligand exchange process. Fourier transform infrared spectroscopy (FTIR) analysis of AuNPs after modification also revealed changes in the waveforms at 2900 cm^-^¹ and 1500 cm^-^¹, further indicating the successful exchange of LA with gold surface ligands (Fig. S2).

Previous studies have suggested that gold nanoparticles with excellent stability and optical sensitivity can, in principle, be obtained first by synthesizing suitably sized nanomaterials, followed by the chemisorption of thiols onto the surface of gold nanoparticles [39]. In this study, LA containing a carboxylate group and a disulfide displaces the citrate and chloride molecules physisorbed on the AuNPs. At high pH, the negative charge of the LA carboxylate group stabilizes the nanomaterials, while the disulfide forms two S-Au bonds that further enhance nanomaterial stability. The morphology and size of AuNPs were determined through hydrodynamic diameter analysis (Fig. S3) and TEM (Fig. S4), which revealed their spherical shape and their approximate size of 12 nm.

### **AuÅPs-AL show biocompatibility and bone-targeting ability**

**Fig. 3:**
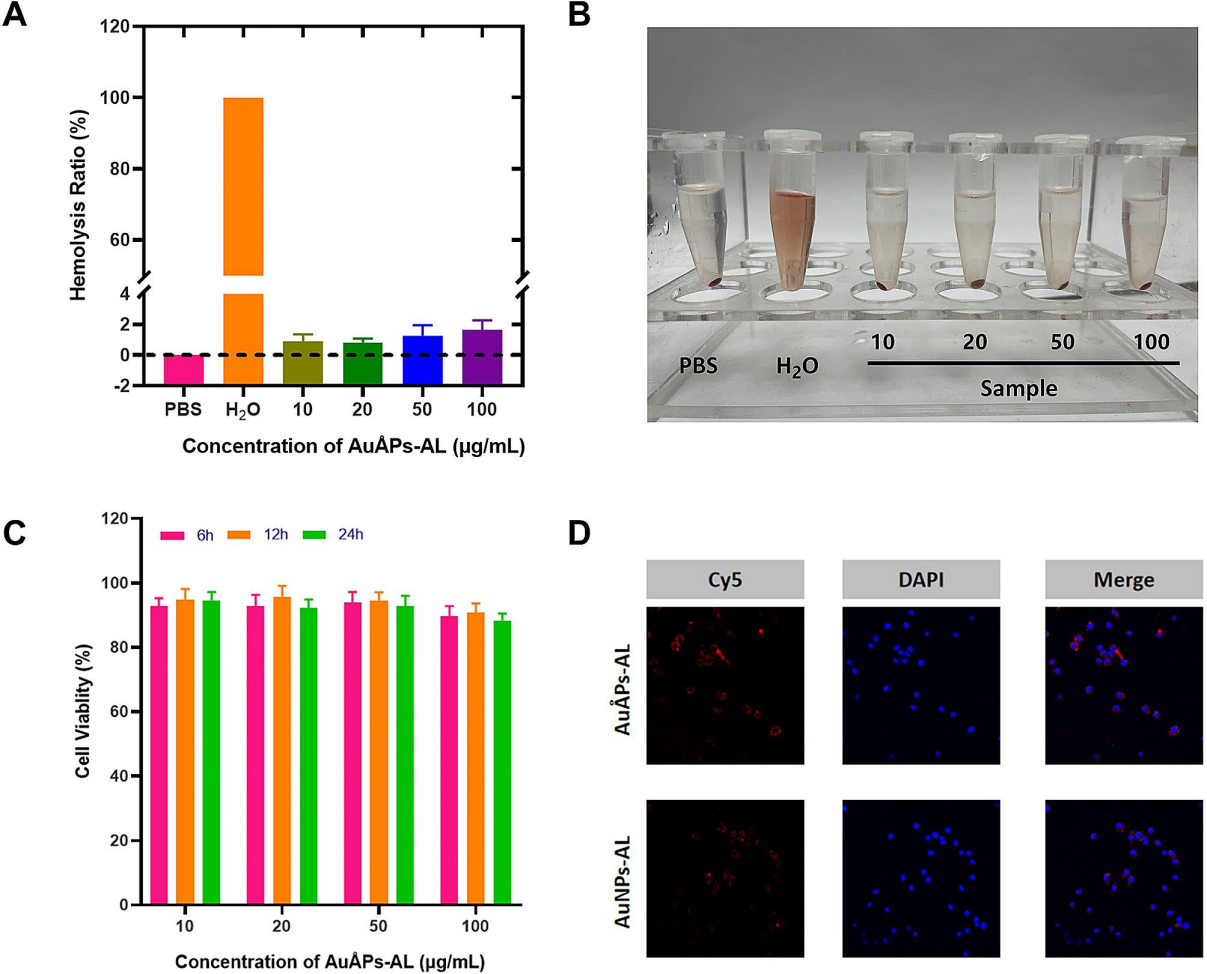
Biocompatibility assessment of AuÅPs-AL. **A** and **B** Hemolysis ratios of different AuÅPs-AL concentrations incubated with RBCs. The data are presented as the mean ± SD (*n* = 3). **C** In vitro cytotoxicity of AuÅPs-AL against Raw 264.7 cells after treatment for 24 h (*n* = 3). **D** Cell uptake capability and cytotoxicity of AuÅPs-AL and AuNPs-AL in Raw 264.7 cells, shown through CLSM images of fluorescence intensity inside Raw 264.7 cells incubated with Cy5-loaded gold particles after 2 h

Because AuÅPs-AL are intended for intravenous administration, their biocompatibility was assessed using a red blood cell (RBC) hemolysis assay, which quantifies the extent to which materials cause cell membrane damage. As depicted in Fig. 3A, the hemolytic activities of AuÅPs-AL were evaluated across various concentrations ranging from 10 to 100 µg/mL. Notably, AuÅPs-AL did not reveal visible hemolytic activity in the range of the tested concentrations. (Fig. 3B). The hemolysis ratios for all tested groups were less than 2%, even at high AuÅPs-AL concentrations reaching 100 µg/mL. The cytotoxicity of AuÅPs-AL within the same concentration range was quantified in Raw 264.7 cells using an 3-(4,5-dimethyl-thiazol-2-yl)-2,5-diphenyl tetrazolium bromide (MTT)  assay, which revealed that cell viability exceeded 90% for all concentration groups over 24 h (Fig. 3C). These findings demonstrate the excellent biocompatibility and negligible cytotoxicity of AuÅPs-AL at concentrations of up to 100 µg/mL, suggesting that they can be safely used as a drug delivery system over a wide concentration range and are suitable for intravenous administration.

An important factor that determines the clinical utility of nanoparticles as a drug delivery vehicle is their ability to enter cells. The cellular uptake capacity of AuÅPs-AL was investigated to understand the influence of ultrasmall particle size on uptake efficiency. Ångstrom-scale (AuÅPs-AL) and nanoscale (AuNPs-AL) gold particles loaded with Cy5 (red) as a fluorescent label were presented to Raw 264.7 cells, and their cellular uptake was analyzed by confocal laser scanning microscopy (CLSM). After 2 h of exposure, both the AuÅPs-AL and AuNPs-AL groups resulted in intense red fluorescence surrounding the cytoplasm of Raw 264.7 cells and accumulated around the nucleus (blue) (Fig. 3D). These findings suggest efficient cellular uptake of gold particles, which was not affected by the ultrasmall size of AuÅPs-AL.

**Fig. 4:**
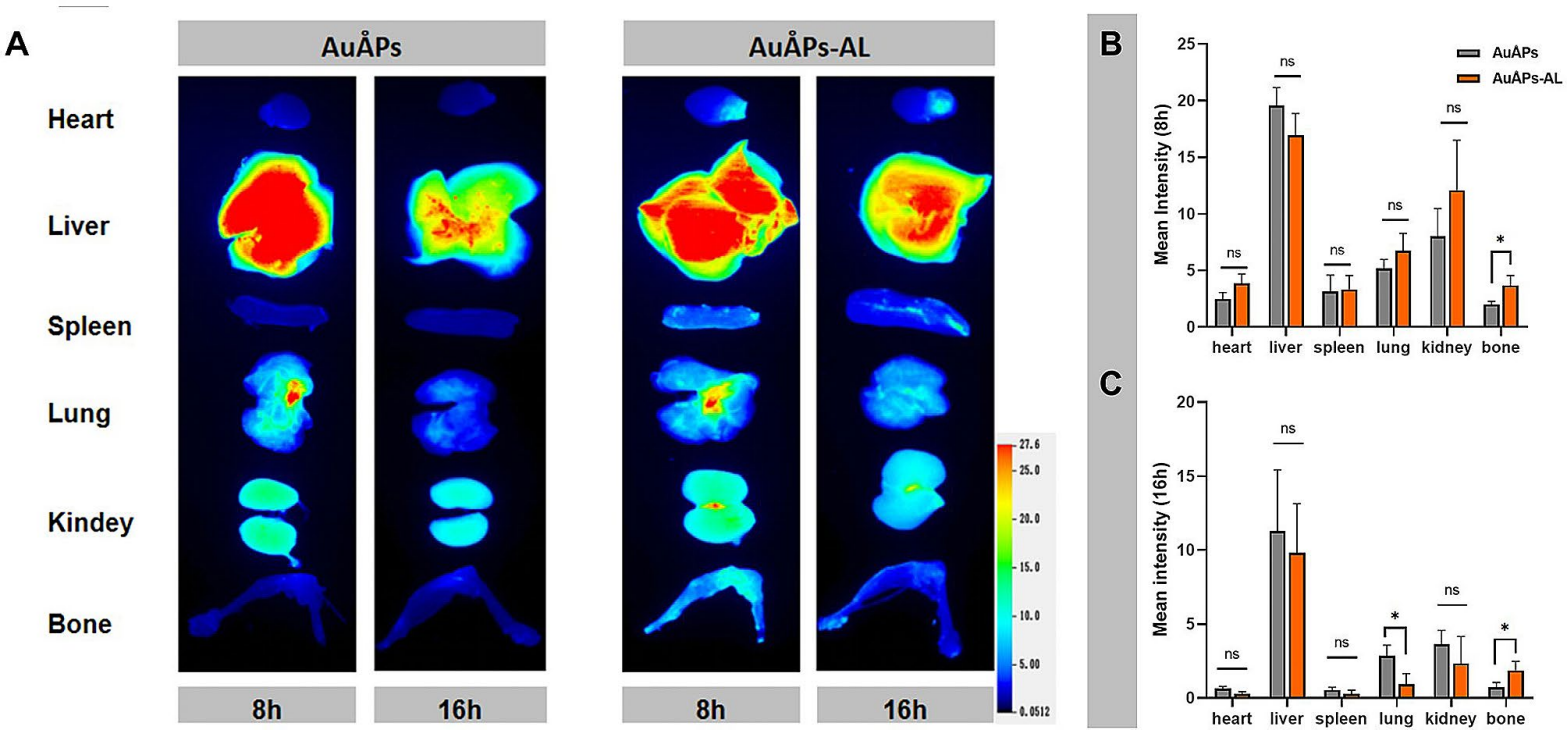
Bone-targeting capability in vivo. **A** Biodistribution of Cy5-labeled AuÅPs and AuÅPs-AL in mice. **B** and **C** Fluorescence intensity of Cy5-labeled AuÅPs and AuÅPs-AL in different organs. Data are presented as the mean ± SD (*n* = 3). ns = not significant; *P < 0.05

The systemic application of nanosized pharmaceuticals has been accompanied by unpredictable side effects [7, 40], which may complicate their use in the treatment of osteoporosis. Bone-targeting gold particles may provide a solution to this problem by potentially minimizing adverse off-target effects and maximizing therapeutic benefits to bone tissue. Common methods for delivering drugs to bone surfaces include using bisphosphonates, acidic oligopeptides, and tetracycline [41]. Considering that bisphosphonates (BPs) can directly modulate bone metabolism and suppress osteoclast activity while having great in vivo bone-targeting ability [42], we hypothesized that combining AuÅPs with bisphosphonates might have a synergistic effect in osteoporosis treatment. Thus, we chose AL as a common anti-osteoporosis drug to act as the bone-targeting group [43, 44]. The AuÅPs were chemically coupled with AL to form AuÅPs-AL, and their in vivo bone-targeting ability was determined following intravenous injection into mice. The fluorescence intensity of Cy5-loaded gold particles with or without AL (Cy5-AuÅPs-AL and Cy5-AuÅPs) was measured by biophotonic imaging at 8 and 16 h after injection to investigate particle biodistribution in major organs, including bones (femur and tibia), heart, liver, spleen, lung and kidney. The fluorescence signals in both AuÅPs groups were comparatively stronger in the liver and kidney than in other locations, likely due to the larger blood circulation of these organs leading to a greater accumulation of gold particles (Fig. 4A-C). Notably, compared to the AuÅPs group, the AuÅPs-AL group showed stronger fluorescence in the tibia and femur, confirming that gold particles conjugated with AL could enable specific accumulation in bone tissue and confer in vivo bone-targeting ability.

### AuÅPs-AL promote in vitro osteogenesis

**Fig. 5:**
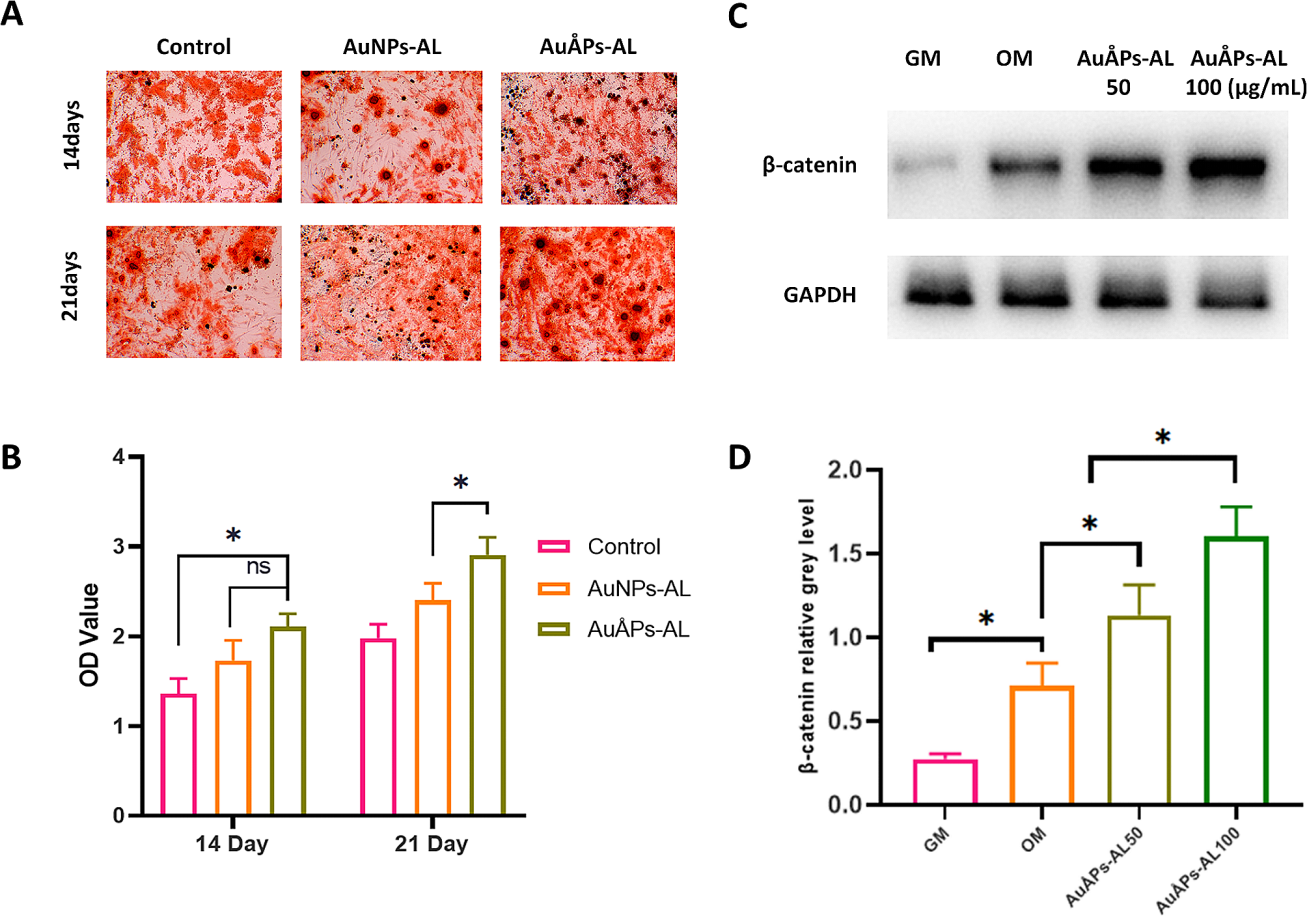
The influence of AuNPs-AL on osteogenic differentiation. **A** and **B** Calcium deposition by MC3T3-E1 cells cultured with AuNPs-AL and AuÅPs-AL, assessed by Alizarin Red staining. Quantitative data are presented as the mean ± SD (*n* = 3). **C** and **D** Western blot analysis showing nonphosphorylated β-catenin protein expression in BMSCs cultured with AuNPs-AL and AuÅPs-AL for 7 days. Quantitative data are presented as the mean ± SD (*n* = 3). ns = not significant; *P < 0.05

A critical parameter for determining the feasibility of new nanomaterial solutions for application in osteoporosis therapy is their ability to induce osteogenesis, which may be tested by in vitro osteogenic induction. Mineralized nodules are considered the final stage markers of osteogenic differentiation [45]. Calcium deposits resembling mineralization typically reach their maximum quantity after in vitro osteogenic induction for two to three weeks [46]. Mineralization was assessed by Alizarin Red staining after MC3T3-E1 cells were cultured with AuNPs-AL and AuÅPs-AL in osteogenic medium for 14 and 21 days (Fig. 5A). All groups, including the control, showed continuous increases in the amount of calcium deposition (red) from 14 to 21 days, but the groups cultured with gold particles exhibited higher mineralization than the control at both time points. Notably, quantitative analysis of Alizarin Red staining revealed significantly higher optical density (OD) values in the AuÅPs-AL group than in the AuNPs-AL group at 21 days (Fig. 5B), suggesting that ultrasmall gold particles could enhance osteogenic differentiation compared to nanoscale gold particles. Other studies have similarly revealed that gold nanoparticles of different sizes can have contrasting effects on cell growth and osteogenesis [47]. For instance, ultrasmall gold nanoclusters with an average diameter of 2.0 nm, synthesized by using lysozyme as a protective template, were found to better promote osteogenic differentiation and reduce osteoclast activity compared to gold nanoparticles [7].

Given the key load-bearing role of bone tissue, the Wnt/β-catenin signaling pathway has been identified as a key player in osteoblast differentiation [48, 49]. Activation of the Wnt/β-catenin pathway inhibits adipogenic differentiation in human adipose-derived mesenchymal stem cells and promotes a shift in cell fate from adipocytes to osteoblasts [50]. We also identified β-catenin as a crucial regulator of osteogenic induction in the presence of AuÅPs-AL through Western blotting. Murine BMSCs cultured for 7 days in osteogenic medium showed a proportional increase in β-catenin protein expression in the presence of higher concentrations of AuÅPs-AL (Fig. 5C and D). This increase in nonphosphorylated β-catenin protein leads to its greater translocation into the cell nucleus, resulting in Wnt/β-catenin pathway activation that eventually promotes osteogenesis by regulating KEY mRNA expression and mineralization [51]. Similar signaling patterns have been reported elsewhere, as chitosan-conjugated gold nanoparticles were also shown to promote bone formation by modulating the Wnt/β-catenin signaling pathway [8]. Our in vitro findings suggest that the Wnt/β-catenin pathway plays a key regulatory role in the enhancements that occur when cells are cultured in the presence of AuÅPs-AL.

### AuÅPs-AL improve bone microarchitecture and strength in osteoporotic mice

**Fig. 6:**
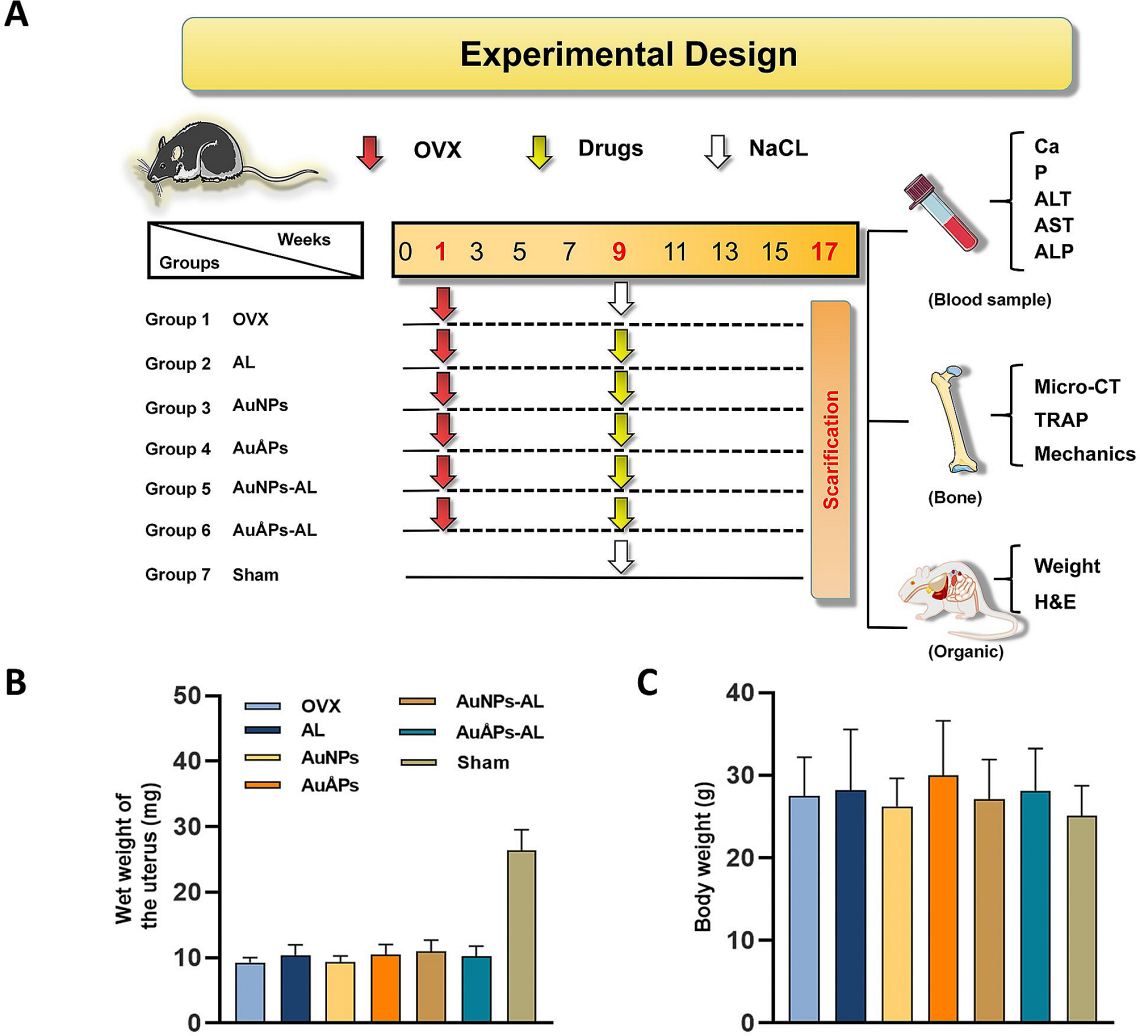
Establishment of an osteoporotic mouse model by OVX. **A** Overview of the treatment protocol. **B** Uterine wet weights in different groups at 17 weeks after treatment. **C** Body weights at 17 weeks after treatment. Data are presented as the mean ± SD (*n* = 6)

A defining characteristic of osteoporosis is trabecular osteopenia. Hence, the microarchitecture of trabecular bone can be used to predict osteopenia and the deterioration of overall bone quality [52]. A mouse model of osteoporosis was established through bilateral OVX of mice to evaluate the effects of AuÅPs-AL in preventing in vivo bone loss. The OVX-induced osteoporosis mouse model is a preclinical model that has been extensively used to mimic the degeneration of bone microstructure, bone loss, and increased bone fragility in osteoporosis [53]. Trabecular bone loss in OVX mice is due to estrogen deficiency and subsequent increased bone turnover, ultimately contributing to osteoporosis. The in vivo study design and treatment plan are illustrated in Fig. 6A. Successful model establishment was confirmed by measurements of uterine wet weight and final body weight (Fig. 6B). The uterine wet weights of all OVX-induced groups were approximately 30% of that of the sham group, indicating successful simulation of low estrogen levels similar to those observed in postmenopausal women. The mice were then intravenously administered PBS, AL, AuNPs, AuÅPs, AuNPs-AL or AuÅPs-AL every three days for 8 weeks prior to analyses.

**Fig. 7:**
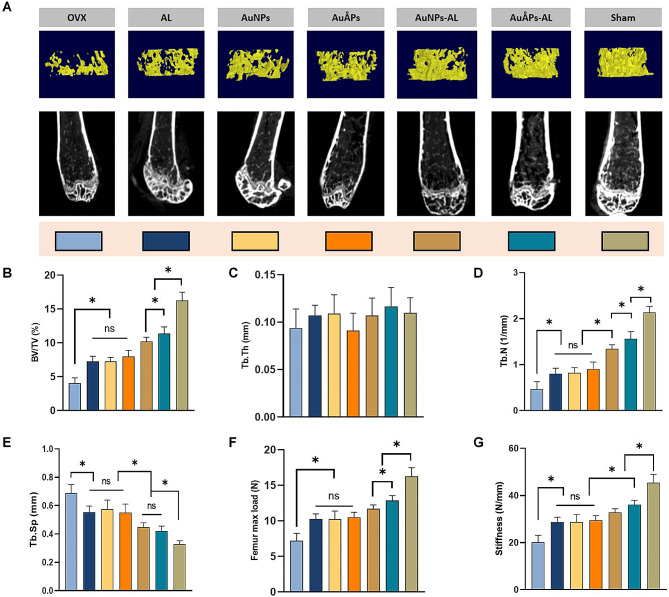
Treatment effects of different groups of gold particles in alleviating in vivo bone loss in an OVX-induced osteoporotic mouse model. **A** Representative micro-CT images showing the three- and two-dimensional microarchitectures of trabeculae in the distal femurs of mice. **B-E** Percent bone volume (BV/TV), trabecular thickness (Tb.Th), trabecular number (Tb.N) and trabecular separation (Tb.Sp) as measured by micro-CT. **F** and **G** Quantitative analysis of bone mechanical strength by the three-point bending test. Data are presented as the mean ± SD (*n* = 6). Ns = not significant; *P < 0.05

Bone quality has long been established as an important indicator for evaluating osteoporosis and bone-related diseases. Micro-computed tomography (Micro-CT) analysis was performed on bone explants to evaluate the microarchitecture of the distal femur (Fig. 7A-E). The OVX group exhibited declines in percent bone volume (BV/TV), trabecular thickness (Tb.Th) and trabecular number (Tb.N) and an increase in trabecular separation (Tb.Sp). These findings collectively suggest the occurrence of bone loss and its deterioration. Compared to nontargeted groups treated with gold particles not containing AL (AuNPs and AuÅPs), the AuÅPs-AL and AuNPs-AL groups exhibited better trabecular bone microarchitecture, including higher BV/TV and Tb.N and lower Tb.Sp. Among all groups, the AuÅPs-AL group showed trabecular bone microarchitecture with parameter values closest to those observed in the sham group. The addition of AL alone without gold particles had a similar effect as the AuNPs and AuÅPs groups, where these three groups all had better trabecular microarchitecture than the OVX group, with no significant differences among them. Combining these findings, the Ångstrom-scale gold particles appear to have had a synergistic effect with the addition of AL in preserving trabecular architecture compared with either component alone. Bone strength is a mechanical property index dependent on bone structure and bone mass. Three-point bending tests were applied to samples to assess the mechanical properties of the mouse right femur. The femur was chosen for analysis because it is the largest bone in the mouse and best approximates a cylindrical cross-section. The maximum load and stiffness measurements exhibited similar trends as that observed for trabecular microarchitecture, where the AL, AuNPs and AuÅPs groups showed improved values compared to the OVX group, while higher values were seen in the AuNPs-AL and AuÅPs-AL groups, with the latter having the closest match to the sham group (Fig. 7F and G). Several studies have reported similar findings [54, 55], suggesting that gold nanoparticles can enhance bone mineral density in osteoporotic mice, mitigate bone loss in OVX-induced osteoporosis, and affect bone metabolism in vivo. Furthermore, positive effects in maintaining bone strength and reducing bone fragility were demonstrated. However, studies using ultrasmall-sized gold nanoparticles (< 3 nm) in bone repair are extremely limited [7], where osteogenic effects have only been shown using cellular models while in vivo efficacy at preventing bone loss has never been explored. In this study, we for the first time tested the effects of AuÅPs using an in vivo model of bone loss/repair, with our data confirming a possible synergistic effect between AuÅPs and AL in preserving bone strength and reducing fracture risk in osteoporosis.

**Fig. 8:**
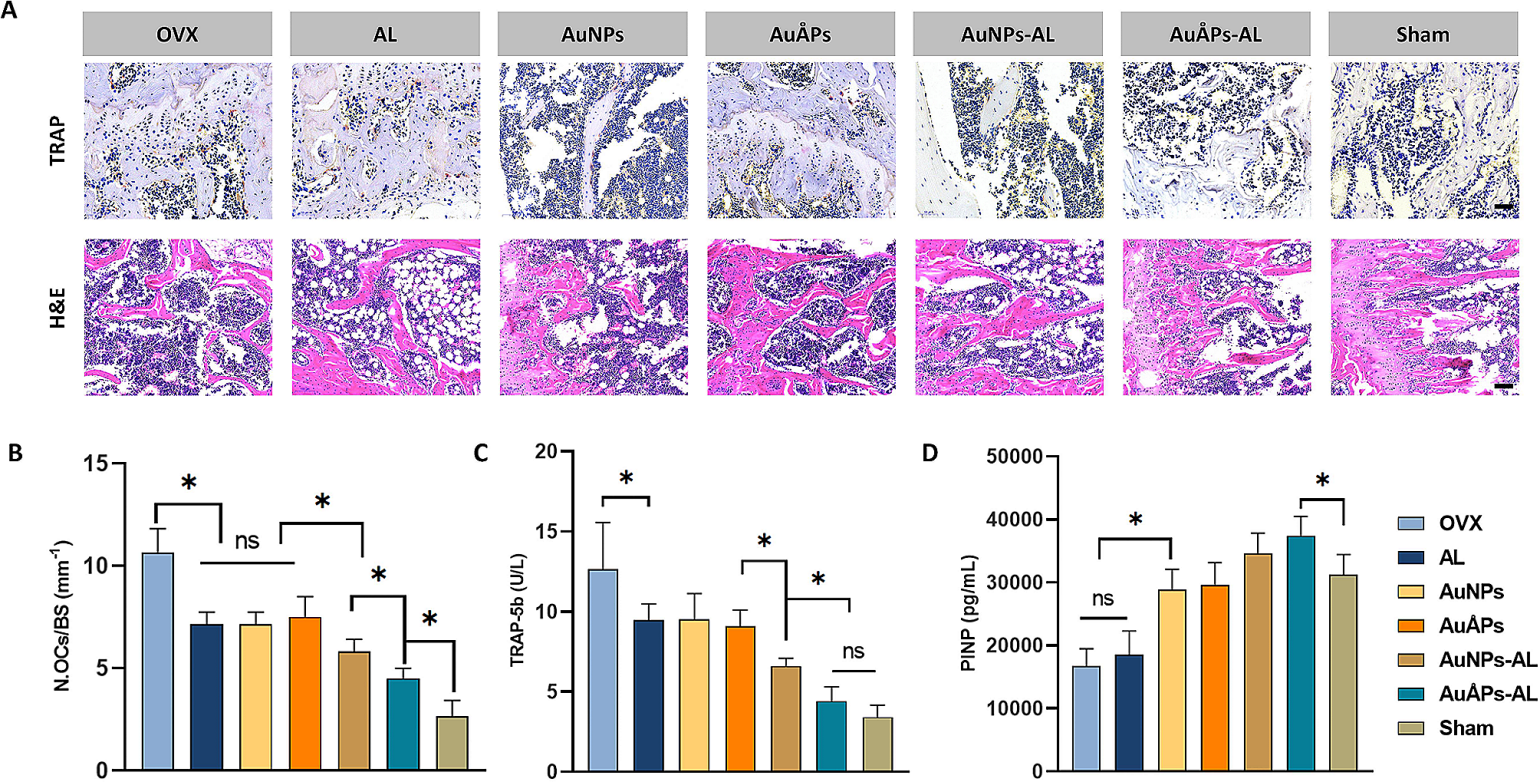
Evaluation of bone microarchitecture by histomorphometry analysis. **A** Representative images of tibial sections stained with TRAP (scale bar = 50 μm) and H&E (scale bar = 100 μm). **B** Quantification of the number of osteoclasts (N. Ocs) from TRAP-stained images. **C** and **D** Effects of treatment groups on serum TRAP-5b and PINP at the end of the study (week 17, *n* = 5). Data are presented as the mean ± SD. Ns = not significant; *P < 0.05

In addition to bone density, the relative contents and properties of organic bone matrix and bone minerals are closely associated with mechanical characteristics and bone strength. Explanted tibia specimens were subjected to histological analysis through hematoxylin and eosin (H&E) and tartrate-resistant acid phosphatase (TRAP) staining to evaluate osteoclast formation and morphologic changes in the trabecular bone (Fig. 8A). Qualitative and quantitative analyses of TRAP staining indicated a marked increase in osteoclasts in the OVX group, which was significantly reduced in the AL, AuNPs and AuÅPs groups and further reduced in the AuNPs-AL and AuÅPs-AL groups, with the AuÅPs-AL group exhibiting a significantly lower osteoclast count than all other treatment groups (Fig. 8B). These results suggest that AuÅPs-AL could suppress osteoclast formation and the bone-resorbing activity of osteoclasts in vivo. Subsequent H&E staining confirmed osteoporotic changes in OVX mice, manifested as an increase in the intertrabecular space accompanied by thinning of bone trabeculae. These changes were effectively mitigated in the groups treated with gold particles, with the AuÅPs-AL group showing the best effects at increasing trabecular density and ameliorating bone loss in vivo. These findings collectively suggest that AuÅPs-AL can exert a bone protective effect in an in vivo osteoporosis model, effectively inhibiting osteoclast formation and deterioration of the trabecular structure.

Changes in the serum levels of bone turnover markers may be used as a complementary indication of the therapeutic effects of osteoporosis treatments, which are more sensitive and easily measured than bone mineral density. Markers such as tartrate-resistant acid phosphatase 5b (TRAP-5b) and procollagen type I N-propeptide (PINP) reflect the balance in overall bone formation and resorption, and clinical evidence suggests that increased fracture risk in osteoporosis is generally accompanied by increased serum levels of bone turnover markers [56]. In this study, the serum levels of TRAP-5b and PINP were measured as biochemical indicators of bone metabolism in osteoporotic mice from different treatment groups (Fig. 8C and D). TRAP-5b, an acid phosphatase isoenzyme specifically expressed in osteoclasts, serves as a highly and specific sensitive indicator of bone resorption [56]. PINP is an extracellular metabolite from type I collagen in bone, and its serum levels can be used to reflect osteoblast activity [57]. The serum levels of TRAP-5b and PINP showed similar trends among treatment groups as those observed from micro-CT and histological analyses. Notably, the AuÅPs-AL group exhibited the most significant changes in these markers compared to all other treatment groups, including the lowest TRAP-5b levels and highest PINP levels, both of which matched the levels observed in the sham group. AuÅPs-AL may therefore have sustained effects in increasing bone formation and decreasing bone resorption in osteoporosis, restoring the balance in bone turnover and helping to maintain bone homeostasis.

### AuÅPs-AL can be safely metabolized in vivo

A primary requirement for nanomaterials intended for biomedical applications is their ability to be safely metabolized following in vivo administration. All experimental groups administered gold particles were examined for biochemical parameters associated with toxic side effects. No significant differences in mouse body weight were observed between any of the treatment groups and the control group (Fig. 6C), suggesting that the gold particles do not impact whole-body energy balance or weight regulation. Although BPs can achieve satisfactory curative effects in osteoporosis, the adverse effects of treatment, including mandibular osteonecrosis, atypical fracture and nephrotoxicity, still represent significant problems [58–60]. As previously reported, combining anti-osteoporosis drugs with antioxidants could improve the drugs’ clinical efficacy [61]. In addition, the use of natural antioxidants could also help prevent the in vivo toxicity of gold particles [23, 62]. In our study, we utilized the antioxidant LA as a dispersant and stabilizer to coat AuÅPs, such that AL could be conjugated through LA to the surface of AuÅPs. AuÅPs-AL showed significantly enhanced bone preservation effects compared to uncoated AuÅPs, however, it is difficult to determine the respective contributions of LA and AL to these effects. Future studies would benefit from investigating the specific effects of LA as a standalone treatment for osteoporosis, as well as its capacity to immobilize other types of osteoporosis drugs to the surface of ultrasmall gold nanoparticles.

**Fig. 9:**
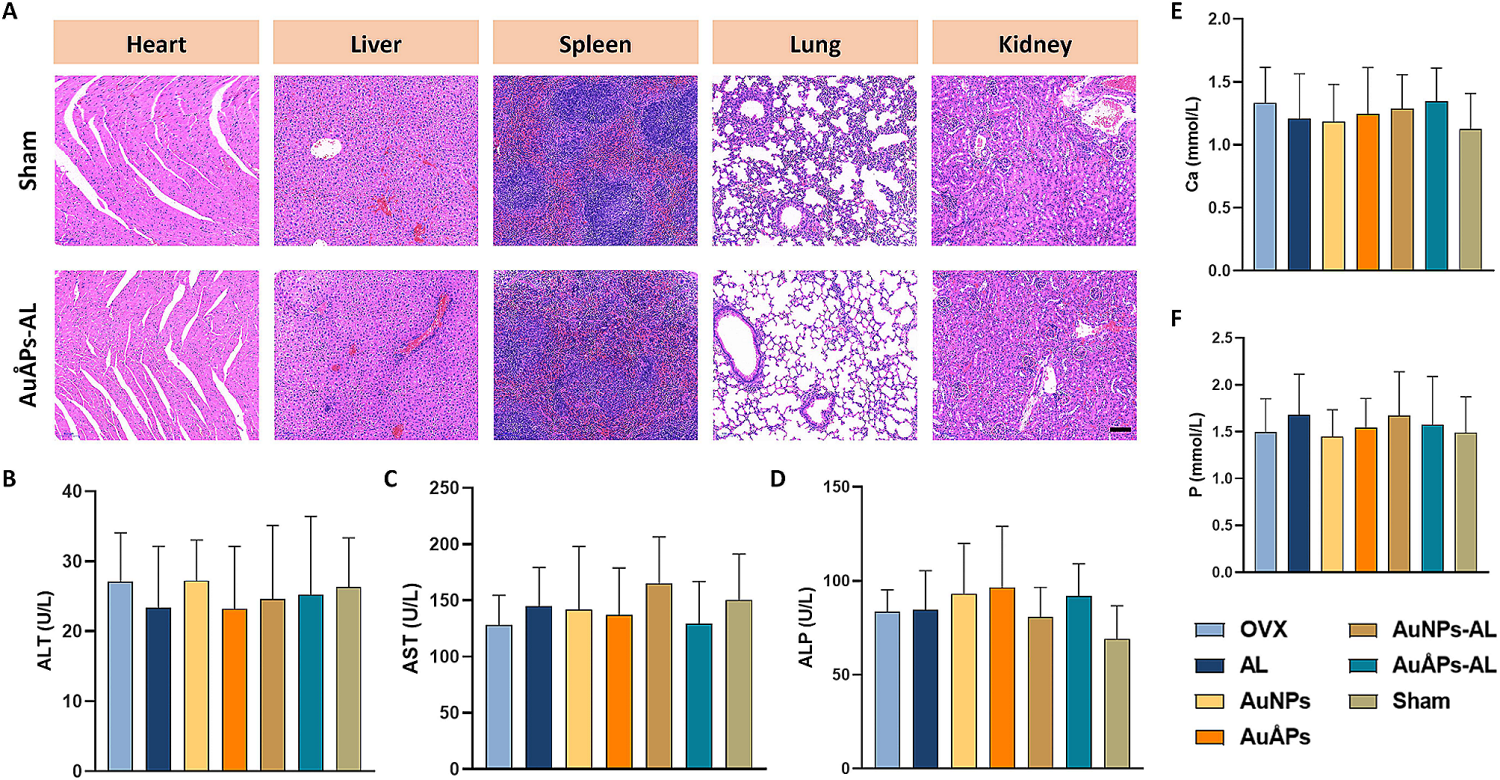
Metabolomic signatures for safety assessment of AuÅPs-AL. **A** Histological evaluation of the heart, liver, spleen, lung and kidney to assess the in vivo metabolism of gold particles. Scale bar = 100 μm. **B-D** Serum levels of ALT, AST, and ALP at the end of the study (week 17, *n* = 5). **E** and **F** Serum levels of Ca and P at the end of the study (week 17, *n* = 5). Data are presented as the mean ± SD (*n* = 5)

Heart, liver, spleen, lung and kidney samples were collected from mice for analysis by H&E staining, which revealed that mice treated with AuÅPs-AL presented no abnormalities or damage in major organs compared to the sham control AuÅPs-AL (Fig. 9A). The results of the hematological study to test serum biochemical parameters associated with in vivo toxicity, including alanine transaminase (ALT), aspartate transaminase (AST), and alkaline phosphatase (ALP), also showed no significant differences when groups treated with gold particles were compared to the control (Fig. 9B-D). In addition, no significant changes in serum calcium (Ca) and phosphorus (P) levels were observed among any of the groups (Fig. 9E and F). These findings confirm that Ångstrom-scale gold particles can be safely metabolized in vivo and do not influence the normal metabolic pathways of Ca and P. Sustained exposure of OVX mice to AuÅPs-AL resulted in minimal toxic effects, suggesting that this system may be safely applied as a potential treatment for osteoporosis.

Given the promising in vivo bone preservation effects observed with AuÅPs-AL as well as the safety of this nanomaterial platform, there is potential to apply it for preventative as well as reactive therapy for osteoporosis. Predictive risk factors for osteoporosis have been analyzed in recent studies [63, 64], which potentially would enable the identification of high-risk patients and justify early intervention with a preventative therapy before clinical presentation of bone deterioration. The possibility of applying AuÅPs-AL as early intervention in patients at risk of developing osteoporosis warrants further investigation in future studies, for instance, by injecting AuÅPs-AL in osteoporotic animal models prior to OVX surgery. In the current study, treatment in osteoporotic mice was administered every 3 days consecutively for a period of 8 weeks after sudden drop in estrogen levels induced by OVX surgery. In this model, we showed that AuÅPs-AL could reverse bone loss and combat symptoms of osteoporosis when administered early, starting at the same time as estrogen decline. Clinical studies have also confirmed that the early stages of menopause or osteoporotic bone loss are considered the best timing for pharmacological interventions [3, 65]. Given the inter-species differences between mice and humans, with the former having a much shorter lifespan and rapid response to treatment, our current data suggest that future clinical application of AuÅPs-AL in osteoporosis treatment might derive the most benefit from early application during the early stages of disease, as well as repeated application possibly every few months. The dosage and frequency of treatment would be minimized for an individual patient due to the high efficiency of drug delivery and therapeutic potency of this AuÅPs-AL system, and could be adjusted according to the severity of symptoms and patient response.

## Conclusions

This study is the first report of a nanomaterial-based system that combines Ångstrom-scale gold particles, an antioxidant, and a bisphosphonate for the treatment of osteoporosis. These components were found to exert a synergistic effect in promoting in vitro osteogenesis and protecting against in vivo bone loss. The AuÅPs-AL developed in this study showed excellent stability, biocompatibility, and bone-targeting ability. They could be efficiently internalized into cells, subsequently accelerating the process of osteogenesis partly through activation of the Wnt/β-catenin signaling pathway. In an established osteoporotic mouse model, AuÅPs-AL preserved bone microarchitecture and strength and protected against bone resorption with minimal adverse effects on nonskeletal tissues. Our study not only highlights the broad prospects of AuÅPs-AL in future biomedical applications relating to bone tissue engineering but also introduces the modification of ultrasmall gold particles as a new direction for developing biocompatible, efficient, and tunable drug delivery systems for disease prevention and treatment.

## Materials and methods

### Materials

All chemicals were obtained from commercial sources and used as received. Milli-Q water (Millipore) with a resistivity of 18.2 MΩ.cm was used in all experiments requiring water. HAuCl_4_ and LA were purchased from Aladdin (China). EDC, NHS, AL, and MTT was purchased from Sigma‒Aldrich (St. Louis, MO, USA). Fetal bovine serum (FBS), α-MEM, and DMEM were obtained from Gibco BRL (Carlsbad, CA, USA). All other solvents of analytical grade were acquired from Sinopharm Chemical Reagent Co., Ltd (Shanghai, China).

### Cells and animals

The mouse macrophage line Raw 264.7, mouse preosteoblast cell line MC3T3-E1, and BMSCs were obtained from Procell Life Science & Technology (Wuhan, China). MC3T3-E1 cells were cultured in α-MEM supplemented with 10% FBS, 100 IU/mL penicillin and 100 µg/mL streptomycin. RAW264.7 cells were cultured in DMEM supplemented with 10% FBS, 100 IU/mL penicillin and 100 µg/mL streptomycin. Mouse BMSCs were cultured in mouse MSC Medium (Cyagen) according to the manufacturer’s protocol (Nuwacell, Hefei, China). The cell cultures were maintained in an incubator at 37 °C in a humidified atmosphere with 5% CO2.

C57BL/6J female mice of specific pathogen-free (SPF) quality were purchased from the Model Animal Research Center of Tongji Medical College of Huazhong University of Science and Technology (HUST, Wuhan, China). All mice were housed under controlled conditions, which included room temperature of 25 °C, humidity maintained at 60 ± 10%, and a natural light–dark cycle throughout the study. All animals were treated according to the regulations of Chinese law and the local Ethics Committee. All animal experiments were reviewed and approved by the IACUC of HUST (IACUC Number: 3469).

### Synthesis of AuÅPs and AuÅPs-AL

To synthesize LA-functionalized AuÅPs, R-LA (1.5 mM) was dissolved in ultrapure water to form a solution under aggressive supersonication, and NaOH was used to adjust the pH values (pH 11) of the solutions for better solubility. Subsequently, a solution of HAuCl_4_ (50 mM) was introduced, resulting in a final Au: LA mole ratio of 1:3 while maintaining vigorous stirring. The mixed solution was heated in an oil bath to 70 °C for 8 h. The resulting solution was centrifuged for 15 min at a rotational speed of 16,000 rpm to remove large particles. Dialysis was continued for 48 h using a dialysis bag (1000 Da) to remove free ions and agents. Following this purification, Ångstrom-scale gold particles were obtained. EDC and NHS were added to the solution and stirred continuously for 1 h, followed by the addition of AL. By utilizing a coupling reaction between the carboxyl group from LA and the amino group from AL in the presence of EDC and NHS, AuÅPs-AL derivatized with the desired compounds were obtained. These derivatives were easily purified through dialysis, as unreacted amines and the coupling agent freely passed through the dialysis membrane, while AuÅPs-AL did not. The obtained particles were subjected to freeze drying and used for all subsequent experiments.

### Characterization of AuÅPs and AuÅPs-AL

TEM was used to observe the particle size, structure, and morphology of AuÅPs or AuÅPs-AL. In short, gold particles at a concentration of 500 ng/µL were permitted to adsorb onto carbon-coated copper grids for approximately 30 min, after which the grids were rinsed in water. TEM imaging was then performed (H-7000FA, Hitachi, Tokyo, Japan). The size distribution of gold particles in aqueous solution was measured by DLS (Nano-ZS90, Malvern Instruments, Malvern, UK). NMR spectra were obtained using a Bruker NMR spectrometer (600 MHz). To measure the stability of AuÅPs-AL in water over 48 h at room temperature, UV and visible light absorption spectra were recorded using a Cary 100 spectrophotometer (Varian, Palo Alto, USA), and fluorescence spectra were recorded using a Fluorolog-3 Spectrofluorometer (HORIBA Jobin Yvon, Edison, USA).

### Synthesis and characterization of gold nanoparticles

Nanoscale gold particles were prepared for comparison purposes using citrate salts as stabilizers and reducing agents. First, a 100 mL solution containing 0.5 mM gold salt in a beaker equipped with a magnetic stirring bar was brought to a boil on a hot plate. Subsequently, 2 mL of a 0.17 M sodium citrate solution was introduced into the solution, followed by 15 min of continuous stirring. The solution underwent a sequence of color transformations, transitioning from light yellow to gray and ultimately settling at a purple‒red color, signifying the completion of the reaction [39, 66]. The electrostatically attached citrate molecules are then replaced by a more stable and covalently bound surface functionalization of LA. After cooling, an excess of LA was introduced into the solution and stirred for 48 h to achieve complete exchange of the citrate molecules. This process of ligand exchange and the amount of ligand do not change the size, shape, or concentration of resulting gold nanoparticles. The attachment of AL to the gold particles was performed as described in Sect. 4.3. The AuNPs and AuNPs-AL were subsequently purified through consecutive centrifugation steps, and the resulting pellets were resuspended in pure water for subsequent analysis. TEM and DLS were used to detect the size and morphology of AuNPs. Ultraviolet wavelength detection and FTIR spectrometer were used to validate the characterization of gold nanoparticles after ligand exchange. Infrared spectra were recorded using an ALPHA-T (Bruker, Germany) instrument within the spectral range of 4000 to 400 cm¹.

### Hemolysis assay

The hemolysis experiments were conducted following a previously established method [67]. Whole blood samples from C57 mice were drawn into tubes containing heparin. The blood was subsequently subjected to centrifugation (4 °C, 3000 rpm, 10 min), yielding RBCs, which were then subjected to three washes with (4 °C) normal saline. Next, the diluted RBCs (2%) were incubated with gold particles at 37 °C for 2 h, after which they were subjected to centrifugation (3000 rpm, 10 min). Absorbance values were read at 540 nm in an EnSpire microplate reader (PerkinElmer, Waltham, MA, USA). PBS served as the negative control, and distilled water was employed as a positive control. The absorbance was measured, and the percent hemolysis was calculated using a previously described formula [31].

### Cytotoxicity assay

Cytotoxicity was assessed using an MTT assay. First, Raw 264.7 cells were cultured with varying concentrations of AuÅPs-AL for 24 h. Subsequently, MTT solution was introduced into the culture medium and incubated for an additional 4 h. DMSO was then used to solubilize the resulting precipitate, and the absorbance values were determined using a microplate reader at OD 570 nm. The cell viability was calculated following the manufacturer’s protocols.

### Cellular uptake assay

To assess the cellular uptake capacity of AuÅPs-AL, CLSM was employed (Olympus FV3000, Tokyo, Japan). To prepare Cy5-labeled gold particles, Cyanine 5 (Cy5, Servicebio, Wuhan, China) was added to AuÅPs-AL or AuNPs-AL and swirled for 4 h in the dark. The solution was then dialyzed (1000 Da dialysis bag) for 2 days against Milli-Q water in the dark, which was changed every 8 h to remove the unconjugated Cy5. Cy5-tagged gold particles were then obtained and used for uptake and in vivo studies. Raw 264.7 cells (1 × 10^4^ cells) were seeded into observation dishes for CLSM and incubated to reach 60–80% confluence. The medium was then exchanged for α-MEM containing AuÅPs-AL or AuNPs-AL labeled with Cy5 at 37 °C. After 4 h, the culture was terminated, and the medium was removed. The cells were fixed with 4% paraformaldehyde and counterstained with DAPI, and images were acquired using CLSM.

### In vivo biodistribution study

Each mouse was intravenously injected with Cy5-labeled AuÅPs or AuÅPs-AL at a concentration of 1 mg/mL and a volume of 0.2 mL. Mice were euthanized after different time intervals (8 and 16 h) by administering an excessive amount of pentobarbital sodium. The major organs (heart, liver, spleen, lungs, kidneys and bones) were separated for subsequent analysis using a Bruker Xtreme imaging system (IVIS, Pearl Trilogy, LI-COR, USA). The excised tissues and organs were washed with PBS buffer (3 times), and the distribution of particles was quantitatively assessed through fluorescence imaging.

### Alizarin Red S staining and calcium deposition assay

To perform calcium phosphate ARS staining, MC3T3-E1 cells were cultured with gold particles in 0.5 mL of osteogenic induction medium (10 nM dexamethasone, 50 µg/mL ascorbic acid and 5 mM β-glycerophosphate) at a concentration of 1 mg/mL for either 14 or 21 days. After incubation, the cells were rinsed twice with PBS, fixed at room temperature for 10 min using 4% paraformaldehyde, and subsequently cultured with 0.1% ARS solution at room temperature for 30 min. Following another round of PBS washing, cell images were captured using an optical microscope (Olympus IMT-2, Tokyo, Japan). For the quantitative assessment of calcium deposition, the stained cells were air-dried and then treated with 5% perchloric acid for 30 min. The resulting solution from each well was transferred into a culture plate, and the absorbance was measured at a wavelength of 405 nm using a plate reader [68]. The recorded results were normalized based on the cell count in each well.

### Western blot

Western blot analysis was conducted to evaluate the expression levels of osteogenesis-related proteins in BMSCs cultured with gold particles for 7 days. Cells were collected and lysed using RIPA lysis buffer (Beyotime). Equal quantities of protein from each sample were separated through SDS‒PAGE and transferred onto polyvinylidene difluoride membranes (Bedford, MA, USA). The β-catenin primary antibody used was purchased from Abcam (Cambridge, MA, USA). GAPDH (Abcam) was used as the internal control. Following four washes with Tris-buffered saline, the membranes were exposed to anti-rabbit or anti-mouse secondary antibodies. The protein bands were visualized using a chemiluminescence system (Tanon, Shanghai, China).

### OVX-induced mouse osteoporosis model

Twelve-week-old C57BL/6 female mice were used to create a postmenopausal osteoporosis model after adaptive feeding for one week, according to published protocols [53]. OVX surgery was performed on the mice to induce osteoporosis 8 weeks before treatment. The mice were allocated into various groups through a random selection process, with each group consisting of six mice. All mice were injected with the treatment through the tail vein for a period of 8 consecutive weeks. The groups treated with AuNPs, AuNPs-AL, AuÅPs and AuÅPs-AL received a dosage of 100 µL per injection at a gold particle concentration of 0.5 µg/µL, administered once every three days. The AL group had alendronate (50 µg/kg dissolved in 100 µL normal saline) injected through the tail vein, once every three days [69, 70]. The OVX and sham groups received only 100 µL saline, once every three days. The whole blood and serum samples were subdivided and stored at − 80 °C in a refrigerator until analysis. After euthanizing the mice using an overdose of pentobarbital sodium, the uterus and body weights were measured to determine their wet weights. Additionally, the right femur and tibia were collected for subsequent experiments.

### micro-CT analysis

The removed femurs were fixed in 4% paraformaldehyde for one day and subsequently subjected to scanning using a micro-CT system (Skyscan 1276, Bruker). The scanning parameters were set as follows: 50 kV tube voltage, 400 µA tube current, and 15 μm voxel size. The manufacturer’s software package was used for image processing and data evaluation (Version 1.13). The trabecular region was selected for analysis, which started 0.15 mm below the growth plate and extended proximally for 0.4 mm. The structure image slices were reconstructed into tomographic and three-dimensional images for further analysis. Trabecular bone parameters, such as BV/TV, Tb.Th, Tb.N, and Tb.Sp, were computed from the 3D reconstruction images.

### Three‑point bending test

The mechanical properties of the femur were assessed via a three-point bending test. All bones were carefully cleaned of surrounding tissue and stored in 0.9% NaCl (4 °C). Subsequently, each sample underwent a three-point bending examination until failure, conducted with a Shimadzu AG-5000 A universal material testing machine (Shimadzu Corp., Kyoto, Japan). The distance between the two lower supporting points was set at 12 mm. A metal pole was positioned at the center between the supports, and a consistent loading speed of 5.0 mm/min was maintained throughout the test. From the load‒deformation curve, the following mechanical parameters were recorded: maximum load (N) and stiffness (N/mm).

### H&E staining of tissues

Explanted tibia bone samples and other organs (heart, liver, spleen, lung and kidney) were fixed in cold 4% paraformaldehyde for 24 h. The bone samples underwent decalcification for 3 weeks using 10% ethylenediaminetetraacetic acid (EDTA). The organ samples were impregnated with molten wax, followed by paraffin embedding and sectioning at a thickness of 4–5 μm. H&E staining was performed on these sections. Finally, the prepared slides were examined under a light microscope (Olympus IMT-2, Tokyo, Japan) to evaluate histopathological alterations.

### TRAP staining

The fixation and decalcification of tibia bone sample sections were performed using the same procedure described above. Following decalcification, the samples were embedded in paraffin, sliced into 10-µm thick slices, and subjected to TRAP staining. The trabecular bone area and the number of osteoclasts within selected regions were calculated using ImageJ software.

### Detection of bone turnover markers in serum

Mouse blood samples were collected and centrifuged (3000 rpm, 10 min) to obtain serum samples. The collected blood samples were subjected to centrifugation at 3000 rpm for 10 min to obtain serum. ALP, ALT, and AST levels in serum were measured. Ca and P levels were measured using standard methods. Additionally, the serum samples were analyzed for TRAP-5b and PINP following the manufacturer’s protocols (Colorful Gene Biological Technology Co., LTD, Wuhan, China).

### Statistical analysis

Data are presented as the mean values ± standard deviations (SD). All statistical analyses were performed with GraphPad Prism 8.0 (GraphPad Software Inc.). Student’s t test was performed to compare values between two groups, and one-way analysis of variance (ANOVA) was used to compare three or more groups. A *P* value less than 0.05 was considered to indicate a significant difference between groups.

### Electronic supplementary material

Below is the link to the electronic supplementary material.

Supplementary Material 1

## Data Availability

No datasets were generated or analysed during the current study.

## Abbreviations

LA: alpha-lipoic acid
AuÅPs: Ångstrom-scale gold particles
AuÅPs-AL: alendronate-loaded Ångstrom-scale gold particles
AuNPs: gold nanoparticles
AL: alendronate
BPs: bisphosphonates
ROS: reactive oxygen species
HA: hydroxyapatite
UV: ultraviolet
SPR: surface plasmon resonance
RBC: red blood cell
CLSM: confocal laser scanning microscopy
OD: optical density
OVX: ovariectomy
BMSCs: bone marrow-derived mesenchymal stem cells
H&E: hematoxylin and eosin
TRAP: tartrate-resistant acid phosphatase
PINP: procollagen type I N-propeptide
ALT: alanine transaminase
AST: aspartate transaminase
ALP: alkaline phosphatase
SPF: specific pathogen-free
FTIR: Flourier transform infrared
