# Supplementary material for: Ångstrom-scale gold particles loaded with alendronate via alpha-lipoic acid alleviate bone loss in osteoporotic mice
Source: J Nanobiotechnology. 2024 Apr 30;22:212. doi: 10.1186/s12951-024-02466-9 (PMC11059737; doi:10.1186/s12951-024-02466-9)
Supplement: Supplementary file 1 — Supplementary Material 1 [file 12951_2024_2466_MOESM1_ESM.docx]

**Supplementary Information**

**Ångstrom-scale gold particles loaded with alendronate via alpha-lipoic acid alleviate bone loss in osteoporotic mice**

Weihang Gao^1^, MD; Jiao Jiao Li^2^, MD; Jingyu Shi^3^, MD; Hongbing Lan^4^, MD; Yuanyuan Guo^3^, PhD; Dehao Fu^1^, MD

^1^ Department of Orthopaedics, Shanghai Sixth People's Hospital Affiliated to Shanghai Jiao Tong University School of Medicine, Shanghai, 200233, P. R. China.

^2^ School of Biomedical Engineering, Faculty of Engineering and IT, University of Technology Sydney, Sydney, NSW 2007, Australia

^3^ Department of Pharmacy, Liyuan Hospital, Tongji Medical College, Huazhong University of Science and Technology, Wuhan, China.

^4^ Tongji School of Pharmacy, Huazhong University of Science and Technology, Wuhan, China

**Corresponding Authors**

Dehao Fu − Department of Orthopaedics, Shanghai Sixth People's Hospital Affiliated to Shanghai Jiao Tong University School of Medicine, Shanghai, 200233, P. R. China; orcid.org/0000-0002-9295-1692; Email: [fudehao@sjtu.edu.cn](mailto:fudehao@sjtu.edu.cn)

Yuanyuan Guo − Department of Pharmacy, Liyuan Hospital, Tongji Medical College, Huazhong University of Science and Technology, Wuhan, China. Email: [yuanyuanguo@hust.edu.cn](mailto:yuanyuanguo@hust.edu.cn)


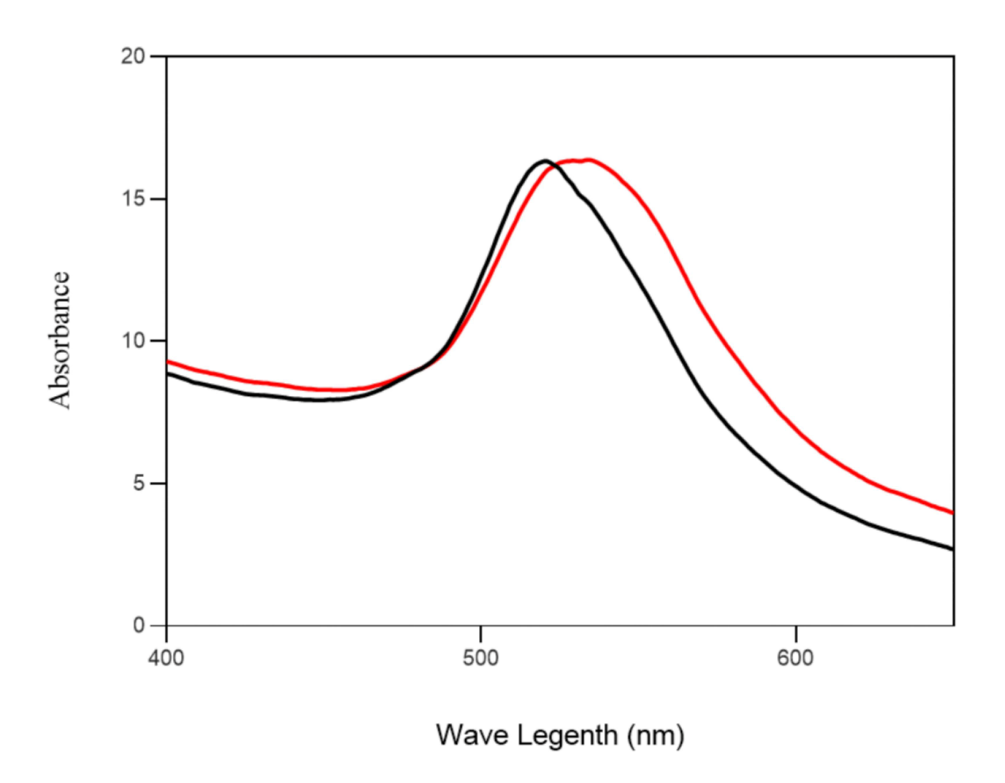


**Fig. S1** UV/vis spectra of citrate (black line) and lipoic acid stabilized (red line) gold nanoparticles. The spectra were obtained in ultrapure water.


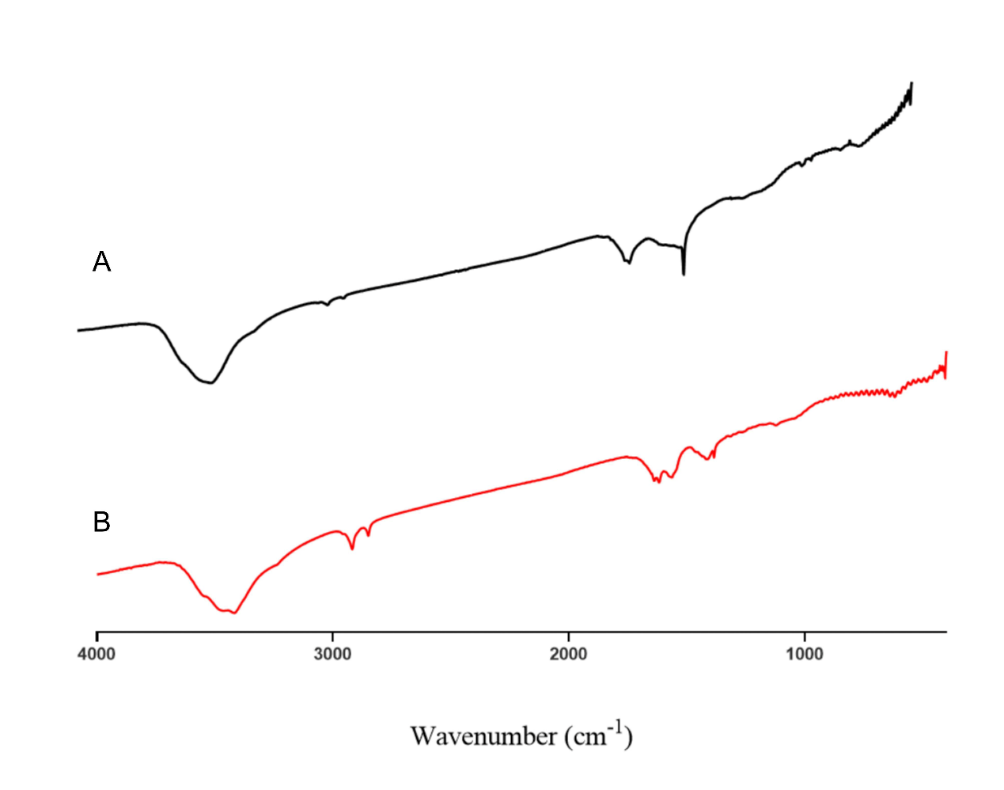


**Fig. S2** Transmission FTIR spectra of citrate (black line) stabilized gold nanoparticles exchanged by lipoic acid (red line).


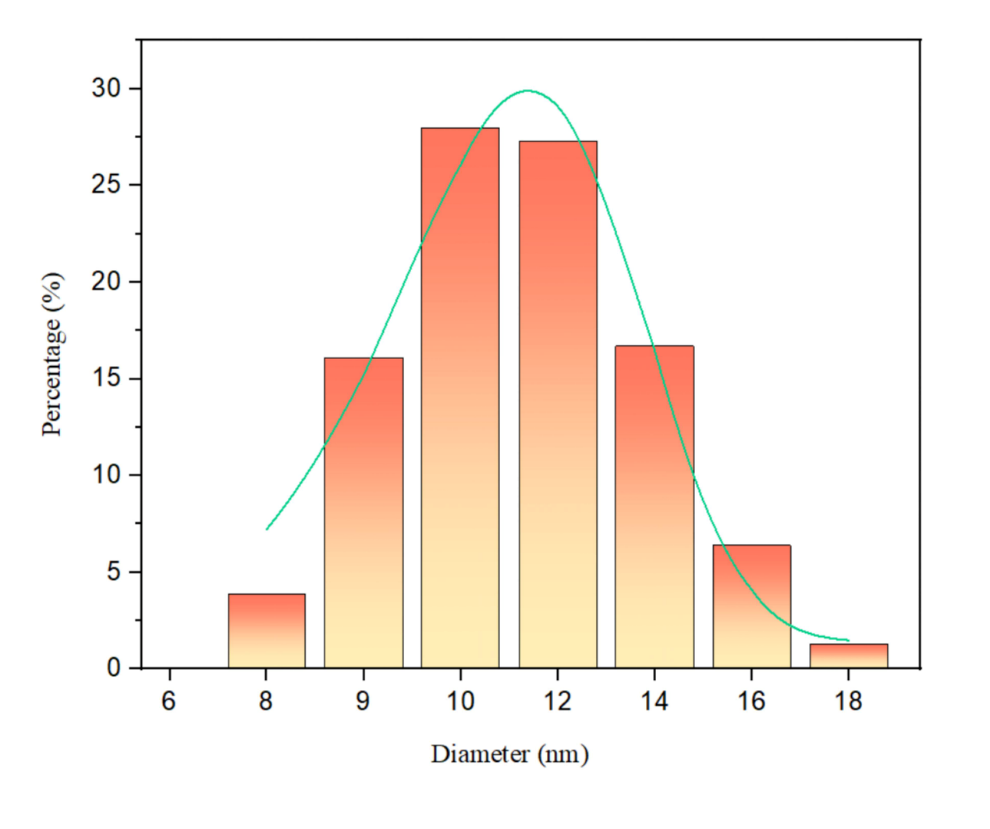


**Fig. S3** DLS spectra of the as-prepared gold nanoparticles in water solution.


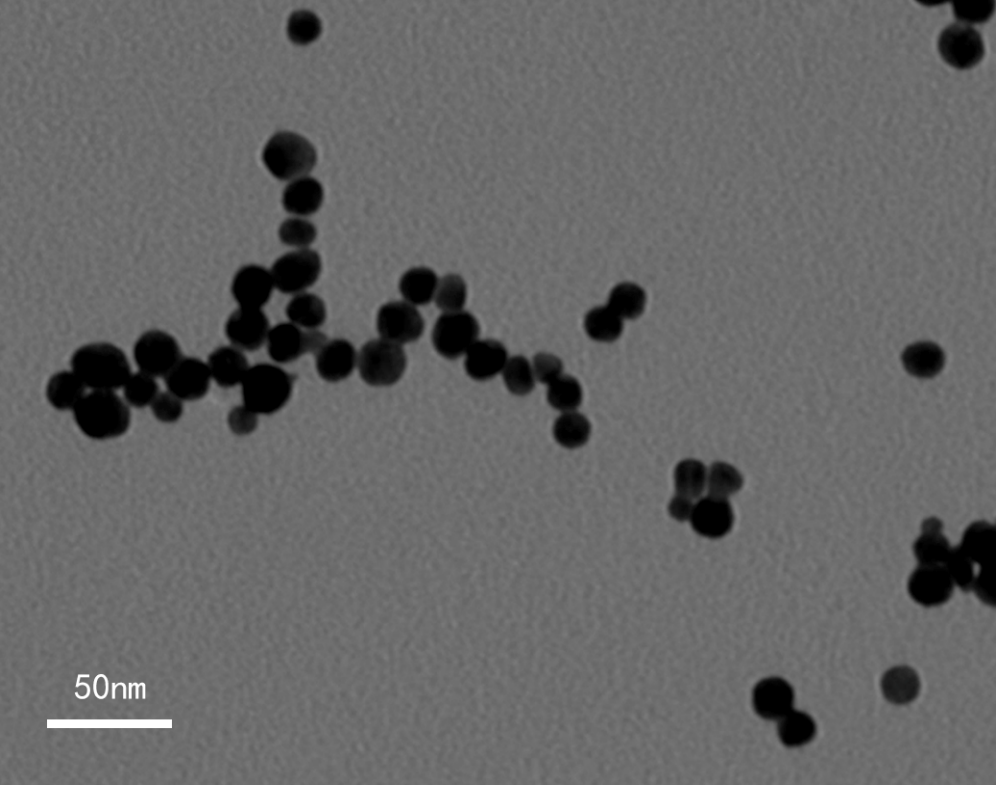


**Fig. S4** The TEM image and core size distribution of the synthesized gold nanoparticles (scale bar=50 nm).


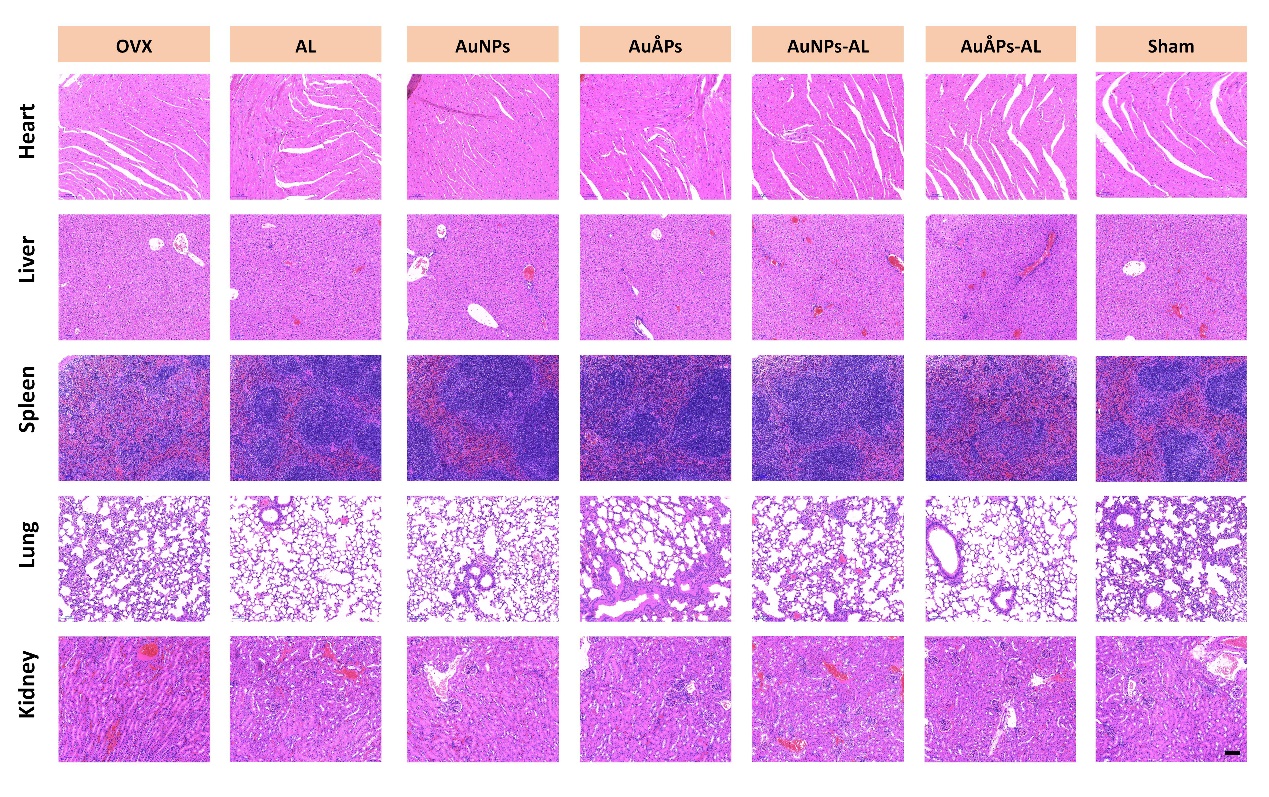


**Fig. S5** Histological evaluation of the heart, liver, spleen, lung, and kidney of mice in OVX, AL, AuNPs, AuÅPs, AuNPs-AL, AuÅPs-AL and sham groups (scale bar=100 μm).
